# High level of fatty liver index predicts new onset of diabetes mellitus during a 10-year period in healthy subjects

**DOI:** 10.1038/s41598-021-92292-y

**Published:** 2021-06-18

**Authors:** Yukimura Higashiura, Masato Furuhashi, Marenao Tanaka, Satoko Takahashi, Masayuki Koyama, Hirofumi Ohnishi, Keita Numata, Takashi Hisasue, Nagisa Hanawa, Norihito Moniwa, Kazufumi Tsuchihashi, Tetsuji Miura

**Affiliations:** 1 Department of Cardiovascular, Renal and Metabolic Medicine, Sapporo Medical University School of Medicine, S-1, W-16, Chuo-ku, Sapporo, 060-8543 Japan; 2 Department of Public Health, Sapporo Medical University School of Medicine, Sapporo, Japan; 3 Department of Health Checkup and Promotion, Keijinkai Maruyama Clinic, Sapporo, Japan

**Keywords:** Diabetes, Metabolic syndrome, Biomarkers

## Abstract

Fatty liver index (FLI), a predictor of nonalcoholic fatty liver disease, has been reported to be associated with several metabolic disorders. This study aimed to evaluate the relationship between FLI and new onset of diabetes mellitus (DM). We investigated the association of FLI with new onset of DM during a 10-year period in subjects who received annual health examinations (n = 28,990). After exclusion of subjects with DM at baseline and those with missing data, a total of 12,290 subjects (male/female: 7925/4365) who received health examinations were recruited. FLI was significantly higher in males than in females. During the 10-year period, DM was developed in 533 males (6.7%) and 128 females (2.9%). Multivariable Cox proportional hazard models with a restricted cubic spline showed that the risk of new onset of DM increased with a higher FLI at baseline in both sexes after adjustment of age, fasting plasma glucose, habits of alcohol drinking and current smoking, family history of DM and diagnosis of hypertension and dyslipidemia at baseline. When the subjects were divided into subgroups according to tertiles of FLI level at baseline (T1–T3) in the absence and presence of impaired fasting glucose (IFG), hazard ratios after adjustment of the confounders gradually increased from T1 to T3 and from the absence to presence of IFG in both male and female subjects. In conclusion, a high level of FLI predicts new onset of DM in a general population of both male and female individuals.

## Introduction

Diabetes mellitus (DM) is one of major medical concerns in metabolic diseases^1^. Because of changes in lifestyle including habits of eating and exercise, the number of patients with DM is continuously increasing worldwide^2^. Since patients with DM have compromised healthy longevity due to multiple complications including diabetic nephropathy and atherosclerotic cardiovascular disease^3,4^, prevention of DM is a critical issue. Therefore, it is crucial to find out subjects at high risk for development of DM for performing appropriate intervention such as exercise encouragement and dietary advice at an early stage.

Nonalcoholic fatty liver disease (NAFLD), a chronic liver disease, has been highlighted as a lifestyle-related disease^5,6^, and the prevalence of NAFLD has been increasing worldwide, leading to a prominent cause of liver-related prognosis^7,8^. It was shown in a cohort study that subjects with NAFLD were at a higher risk for the development of DM than were those without NAFLD^9^. Meta-analyses also showed that NAFLD diagnosed by altered serum liver enzymes, radiological findings or histological evidence increases the risk of type 2 DM^10,11^.

For diagnosis of NAFLD, liver biopsy as an invasive procedure is required^12^, but several noninvasive procedures in adequate concordance with histological findings have recently been established using imaging tools and several biochemical markers including fatty liver index (FLI)^13^. FLI calculated by using waist circumference (WC), body mass index (BMI), and levels of triglycerides and γ-glutamyl transferase (γGTP)^14^ has been reported to be highly concordant with the histological criteria for NAFLD^15–17^. It has recently been reported that NAFLD diagnosed by FLI is a good predictor for incidence of type 2 DM^18–25^ (Supplementary Table S1). However, the relationship of FLI with new onset of DM has not yet been investigated in a large cohort with a sufficiently long observational period and/or as a continuous variable for FLI. Therefore, in the present study, we investigated the effect of FLI level at baseline on new onset of DM during a 10-year period in a large number of subjects divided by sex.

## Results

### Characteristics of the study subjects

**Figure 1:**
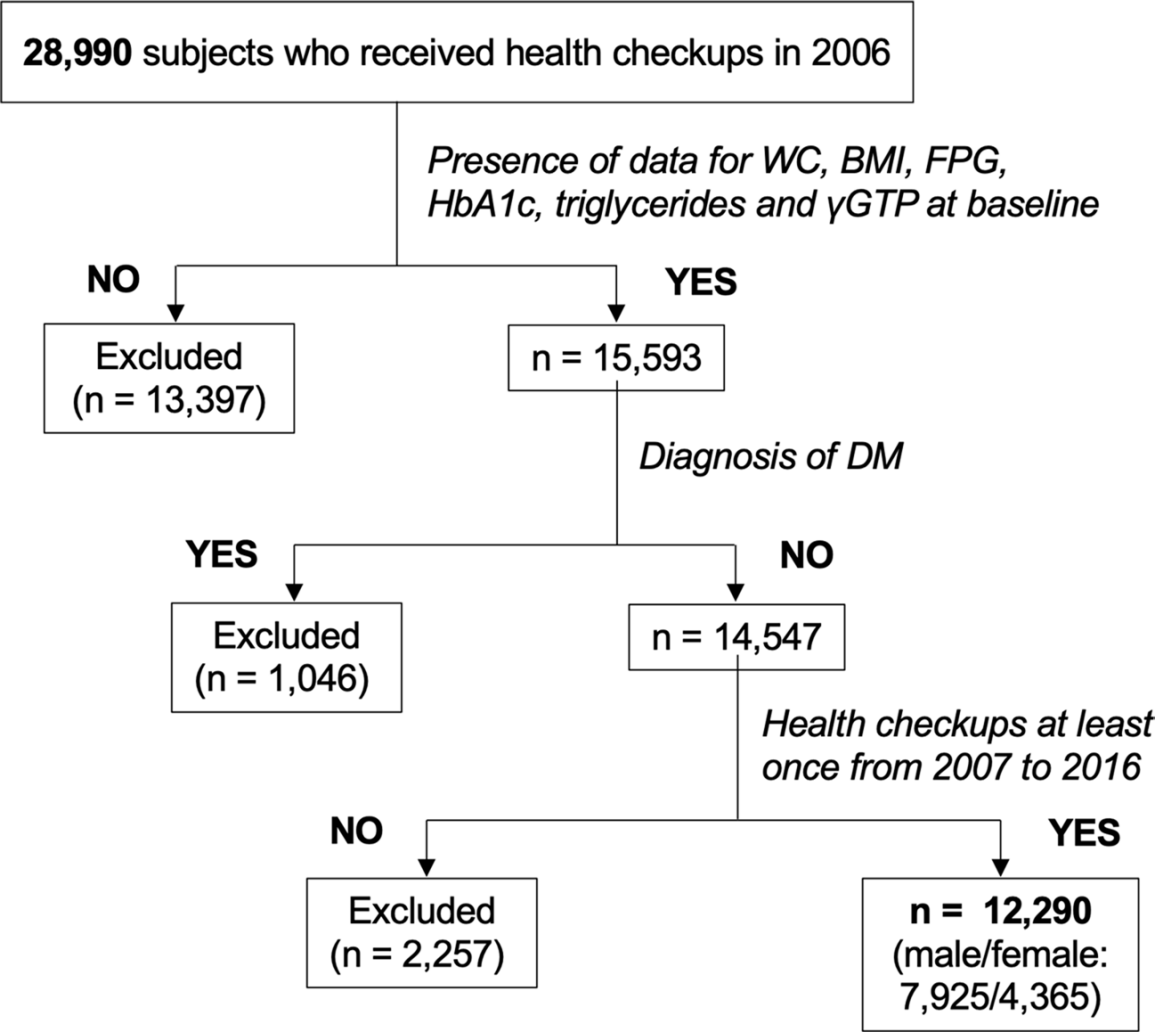
Flow chart of the selected study participants. Among 28,990 subjects enrolled in 2006, a total of 12,290 subjects (male/female: 7925/4365) were finally recruited for analyses in the present study. *BMI* body mass index, *DM* diabetes mellitus, *FPG* fasting plasma glucose, *γGTP* γ-glutamyl transferase, *HbA1c* hemoglobin A1c, *WC* waist circumference.

A flow chart of the study participants is shown in Fig. 1. The characteristics of the enrolled and excluded subjects are shown in Supplementary Table S2. The excluded subjects were significantly younger and more metabolic healthy than the enrolled subjects. Demographic parameters and metabolic profiles of the recruited subjects are shown in Table 1. Male subjects had significantly larger BMI and WC, higher systolic and diastolic blood pressures, higher levels of albumin, uric acid, fasting plasma glucose (FPG), hemoglobin A1c (HbA1c) and triglycerides and lower levels of estimated glomerular filtration rate (eGFR) and high-density lipoprotein cholesterol than did female subjects. FLI was significantly higher in male subjects than in female subjects. The frequencies of habits of smoking and alcohol drinking were lower in female subjects than in male subjects.

**Table 1:** Characteristics of the recruited subjects.

	Total	Male	Female	*P*
	n = 12,290	n = 7925	n = 4365	
Age (years)	48 ± 10	48 ± 10	48 ± 10	< 0.001
Body mass index	23.2 ± 3.4	24.0 ± 3.1	21.7 ± 3.3	< 0.001
Waist circumference (cm)	83.4 ± 9.2	85.9 ± 8.3	79.9 ± 9.1	< 0.001
Systolic blood pressure (mmHg)	117 ± 16	120 ± 16	111 ± 16	< 0.001
Diastolic blood pressure (mmHg)	74 ± 11	77 ± 11	70 ± 10	< 0.001
Smoking habit	4231 (34.4)	3431 (43.3)	800 (18.3)	< 0.001
Alcohol drinking habit	5634 (45.8)	4492 (56.7)	1142 (26.1)	< 0.001
**Family history**				
Diabetes mellitus	2115 (17.2)	1220 (15.4)	895 (20.5)	< 0.001
**Comorbidity**				
Hypertension	2068 (16.8)	1582 (20.0)	486 (11.1)	< 0.001
Dyslipidemia	2720 (22.1)	1674 (21.1)	1046 (24.0)	< 0.001
**Biochemical data**				
Albumin (g/dL)	4.4 ± 0.2	4.4 ± 0.2	4.3 ± 0.2	< 0.001
eGFR (mL/min/1.73 m^2^)	84.5 ± 14.6	83.3 ± 14.0	86.7 ± 15.4	< 0.001
Uric acid (mg/dL)	5.5 ± 1.4	6.1 ± 1.2	4.4 ± 0.9	< 0.001
FPG (mg/dL)	90 ± 9.3	92 ± 9.3	86 ± 8.4	< 0.001
Hemoglobin A1c (%)	5.2 ± 0.4	5.2 ± 0.4	5.2 ± 0.4	< 0.001
AST (U/L)	23 (18–26)	25 (19–28)	20 (16–22)	< 0.001
ALT (U/L)	26 (15–31)	31 (18–36)	18 (12–20)	< 0.001
γGTP (U/L)	50 (19–56)	63 (26–72)	26 (14–26)	< 0.001
Total cholesterol (mg/dL)	204 ± 34	205 ± 34	204 ± 34	0.05
LDL cholesterol (mg/dL)	122 ± 31	124 ± 31	119 ± 31	< 0.001
HDL cholesterol (mg/dL)	61 ± 16	56 ± 14	69 ± 15	< 0.001
Non-HDL cholesterol (mg/gL)	144 ± 35	149 ± 35	135 ± 34	< 0.001
Triglycerides (mg/dL)	113 (63–136)	131 (77–158)	78 (50–93)	< 0.001
FLI	30.1 (7.9–48.2)	38.6 (16.5–58.2)	14.5 (3.5–17.5)	< 0.001

Variables are expressed as number (%), means ± SD or medians (interquartile ranges).

*AST* aspartate aminotransferase, *ALT* alanine aminotransferase, *eGFR* estimated glomerular filtration rate, *FLI* fatty liver index, *FPG* fasting plasma glucose, *γGTP* γ-glutamyl transferase, *HDL* high-density lipoprotein, *LDL* low-density lipoprotein.

Basal characteristics of male and female subjects divided into the three subgroups according to tertiles of FLI levels at baseline are shown in Tables 2 and 3, respectively. There were significant differences in levels of FPG and HbA1c, prevalence of alcohol drinking habit, comorbidity of hypertension and dyslipidemia, and family history of DM between the three groups of FLI in both male and female subjects. Levels of FPG and HbA1c in the T3 group of FLI tended to be higher than those in the T1 group of FLI in both male and female subjects.

**Table 2:** Characteristics of male subjects divided by tertiles of FLI at baseline (n = 7925).

	T1 (0.9–21.8)	T2 (21.9–49.7)	T3 (49.8–99.7)	*P*
	n = 2650	n = 2633	n = 2642	
Age (years)	47 ± 11	49 ± 9	48 ± 9	< 0.001
Body mass index	21.4 ± 1.9	23.9 ± 1.9	26.6 ± 3.0	< 0.001
Waist circumference (cm)	78.7 ± 5.3	86.1 ± 4.9	93.1 ± 7.0	< 0.001
Systolic blood pressure (mmHg)	115 ± 15	120 ± 15	125 ± 15	< 0.001
Diastolic blood pressure (mmHg)	73 ± 10	77 ± 10	81 ± 10	< 0.001
Smoking habit	1139 (44.3)	1121 (43.5)	1219 (47.5)	0.007
Alcohol drinking habit	1011 (37.6)	1189 (44.2)	1271 (47.4)	< 0.001
**Family history**				
Diabetes mellitus	372 (14.0)	405 (15.3)	443 (16.8)	0.023
**Comorbidity**				
Hypertension	152 (5.7)	299 (11.1)	447 (16.7)	< 0.001
Dyslipidemia	39 (1.5)	113 (4.2)	176 (6.6)	< 0.001
**Biochemical data**				
Albumin (g/dL)	4.4 ± 0.2	4.4 ± 0.2	4.5 ± 0.2	< 0.001
eGFR (mL/min/1.73 m^2^)	85.1 ± 14.0	82.3 ± 13.9	82.6 ± 14.1	< 0.001
Uric acid (mg/dL)	5.7 ± 1.1	6.1 ± 1.2	6.6 ± 1.2	< 0.001
FPG (mg/dL)	89 ± 9	92 ± 9	95 ± 12	< 0.001
Hemoglobin A1c (%)	5.1 ± 0.4	5.3 ± 0.4	5.4 ± 0.4	< 0.001
AST (U/L)	21 (17–23)	24 (19–26)	31 (22–34)	< 0.001
ALT (U/L)	20 (15–24)	28 (19–33)	45 (27–53)	< 0.001
γGTP (U/L)	30 (20–34)	53 (30–61)	108 (51–125)	< 0.001
Total cholesterol (mg/dL)	194 ± 30	206 ± 32	216 ± 35	< 0.001
LDL cholesterol (mg/dL)	116 ± 28	126 ± 30	126 ± 33	< 0.001
HDL cholesterol (mg/dL)	62 ± 15	56 ± 14	51 ± 12	< 0.001
Non-HDL cholesterol (mg/dL)	132 ± 30	150 ± 32	165 ± 35	< 0.001
Triglycerides (mg/dL)	78 (58–94)	120 (87–144)	197 (125–230)	< 0.001

Variables are expressed as number (%), means ± SD or medians (interquartile ranges).

*AST* aspartate aminotransferase, *ALT* alanine aminotransferase, *eGFR* estimated glomerular filtration rate, *FLI* fatty liver index, *FPG* fasting plasma glucose, *γGTP* γ-glutamyl transferase, *HDL* high-density lipoprotein, *LDL* low-density lipoprotein.

**Table 3:** Characteristics of female subjects divided by tertiles of FLI at baseline (n = 4365).

	T1 (0.4–4.4)	T2 (4.5–12.6)	T3 (12.7–97.4)	*P*
	n = 1458	n = 1455	n = 1452	
Age (years)	43 ± 9	48 ± 9	51 ± 10	< 0.001
Body mass index	19.2 ± 1.5	21.2 ± 1.7	24.8 ± 3.3	< 0.001
Waist circumference (cm)	70.9 ± 4.7	78.0 ± 4.8	87.7 ± 7.9	< 0.001
Systolic blood pressure (mmHg)	105 ± 13	110 ± 16	118 ± 16	< 0.001
Diastolic blood pressure (mmHg)	66 ± 9	69 ± 10	75 ± 10	< 0.001
Smoking habit	263 (18.6)	254 (17.9)	285 (20.2)	0.219
Alcohol drinking habit	211 (14.5)	257 (17.5)	261 (17.8)	0.001
**Family history**				
Diabetes mellitus	272 (18.7)	292 (20.0)	331 (22.8)	0.020
**Comorbidity**				
Hypertension	28 (1.9)	76 (5.2)	216 (14.8)	< 0.001
Dyslipidemia	21 (1.4)	53 (3.6)	105 (7.2)	< 0.001
**Biochemical data**				
Albumin (g/dL)	4.3 ± 0.2	4.3 ± 0.2	4.3 ± 0.2	0.234
eGFR (mL/min/1.73 m^2^)	89.1 ± 15.1	85.9 ± 15.3	85.0 ± 15.6	< 0.001
Uric acid (mg/dL)	4.1 ± 0.8	4.3 ± 0.9	4.8 ± 1.0	< 0.001
FPG (mg/dL)	83 ± 7	86 ± 8	90 ± 9	< 0.001
Hemoglobin A1c (%)	5.1 ± 0.3	5.2 ± 0.3	5.3 ± 0.4	< 0.001
AST (U/L)	19 (16–20)	20 (16–22)	22 (18–24)	< 0.001
ALT (U/L)	14 (11–16)	16 (12–18)	23 (15–27)	< 0.001
γGTP (U/L)	16 (12–18)	21 (14–23)	41 (19–44)	< 0.001
Total cholesterol (mg/dL)	191 ± 31	205 ± 32	216 ± 33	< 0.001
LDL cholesterol (mg/dL)	106 ± 26	120 ± 30	131 ± 31	< 0.001
HDL cholesterol (mg/dL)	75 ± 14	70 ± 14	63 ± 14	< 0.001
Non-HDL cholesterol (mg/dL)	117 ± 27	134 ± 31	154 ± 34	< 0.001
Triglycerides (mg/dL)	49 (40–60)	68 (54–85)	100 (76–134)	< 0.001

Variables are expressed as number (%), means ± SD or medians (interquartile ranges).

*AST* aspartate aminotransferase, *ALT* alanine aminotransferase, *eGFR* estimated glomerular filtration rate, *FLI* fatty liver index, *FPG* fasting plasma glucose, *γGTP* γ-glutamyl transferase, *HDL* high-density lipoprotein, *LDL* low-density lipoprotein.

### Cumulative incidence of new onset of DM during the follow-up period

Among the 12,290 subjects (male/female: 7925/4365), 533 male subjects (6.7%) and 128 female subjects (2.9%) developed new onset of DM during a 10-year period. The mean follow-up period was 9.5 years (range: 1–10 years), and follow-up summation was 82,709 (male/female: 53,320/29,389) person-years. The cumulative incidence of new onset of DM was 6.1% (male/female: 7.7%/3.1%).

### Prediction of new onset of DM by levels of FLI and FPG

**Figure 2:**
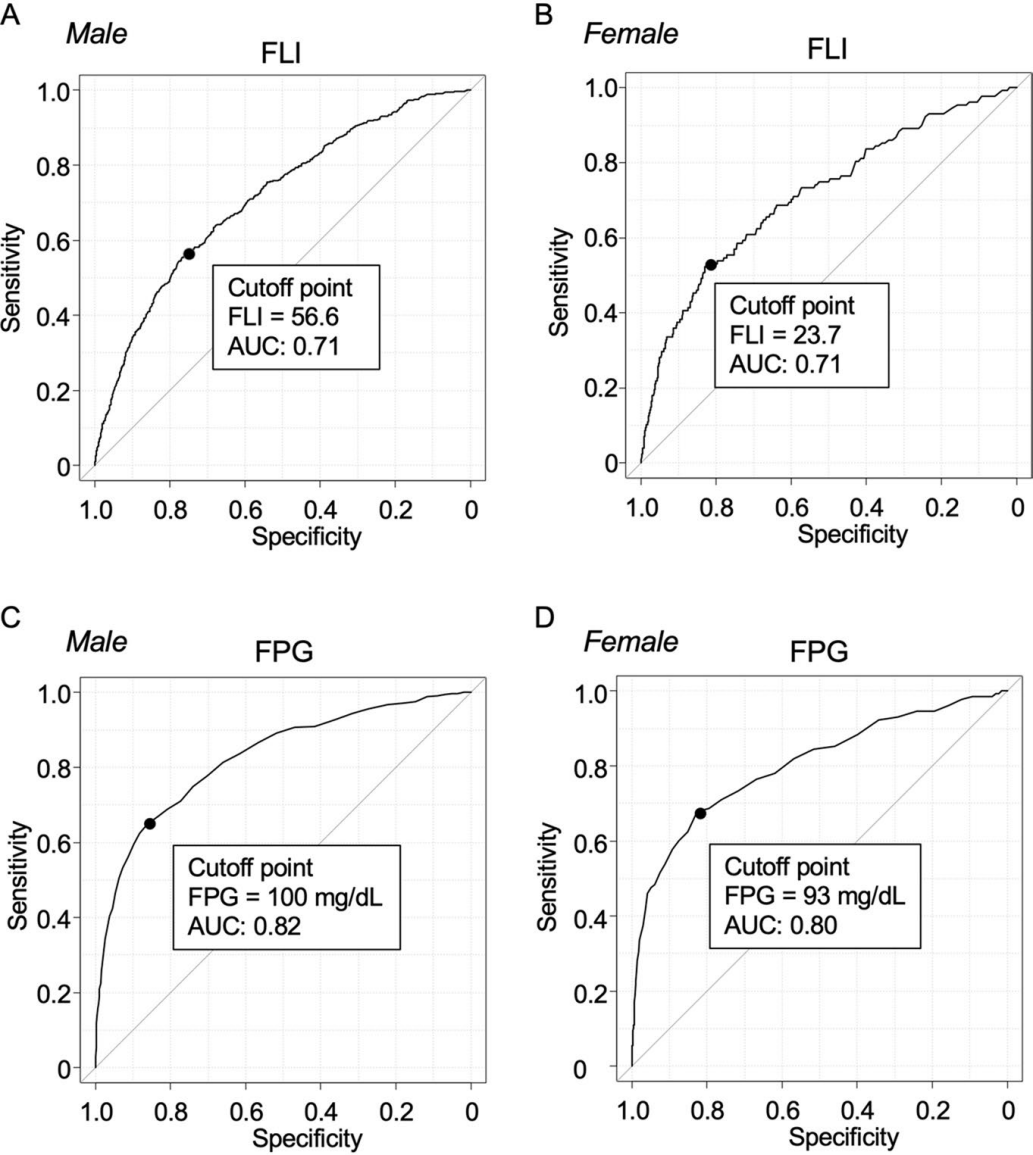
Prediction of new onset of DM by FLI and FPG at baseline. (**A**,**B**) Receiver operating characteristic (ROC) curves of fatty liver index (FLI) at baseline to predict new onset of diabetes mellitus (DM) in males (**A**) and females (**B**). (**C**,**D**) ROC curves of fasting plasma glucose (FPG) at baseline to predict new onset of DM in males (**C**) and females (**D**). *AUC* area under curve, *CI* confidence interval.

Receiver operating characteristic (ROC) curve analyses for predicting new onset of DM showed that the area under curves (AUCs) of FLI at baseline in males (Fig. 2A) and females (Fig. 2B) were 0.71 and 0.71, respectively. The cutoff points of FLI at baseline in males and females were 56.6 and 23.7, respectively. On the other hand, the AUCs of FPG at baseline for predicting new onset of DM in ROC analyses in males (Fig. 2C) and females (Fig. 2D) were 0.82 and 0.80, respectively. The cutoff points of FPG at baseline in male and female subjects were 100 mg/dL and 93 mg/dL, respectively. The AUCs of FPG tended to be higher than those of FLI in both male and female subjects.

### Risk of FLI at baseline for new onset of DM during a 10-year follow-up period

**Figure 3:**
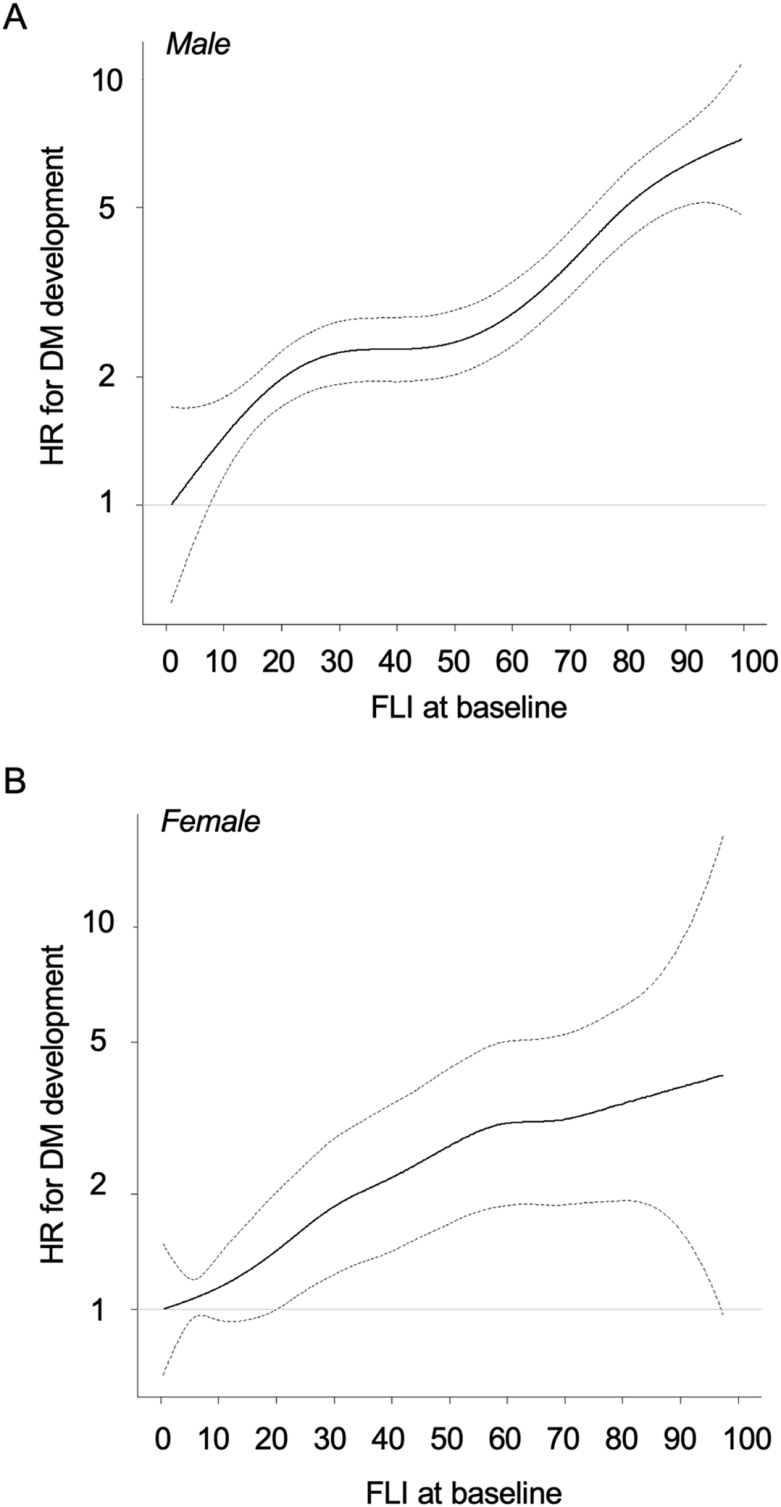
Hazard ratio of the development of DM by FLI at baseline. (**A**,**B)** Hazard ratios (HRs) for the development of diabetes mellitus (DM) by fatty liver index (FLI) at baseline by multivariable Cox proportional hazard models with a restricted cubic spline in males (**A**) and females (**B**) after adjustment of age, fasting plasma glucose, habits of smoking and alcohol drinking, family history of DM, and diagnosis of hypertension and dyslipidemia at baseline during a 10-year follow-up period. Solid line: HR, dashed line: 95% confidence interval (CI). The reference values of FLI in males and females were 0.9 and 0.4 as minimum values, respectively.

Multivariable Cox proportional hazard models with a restricted cubic spline showed that the hazard ratio (HR) of DM development increased with a higher FLI at baseline in both males (Fig. 3A) and females (Fig. 3B) after adjustment of age, FPG, habits of smoking and alcohol drinking, family history of DM and diagnosis of hypertension and dyslipidemia at baseline.

When the 1st tertile (T1) group of FLI was used as the reference, multivariable Cox proportional hazard model analysis after adjustment of age, FPG, habits of smoking and alcohol drinking, family history of DM and diagnosis of hypertension and dyslipidemia showed that HRs in the T2 and T3 groups were significantly higher than HR in the T1 group of FLI in male subjects (Table 4). In female subjects, the adjusted HR in the T3 group of FLI was significantly higher than that in the T1 group of FLI in female subjects (Table 4).

**Table 4:** Multivariable Cox proportional hazard analyses for new onset of DM in tertiles of FLI.

	Male (n = 7925)		Female (n = 4365)	
	HR (95% CI)	*P*	HR (95% CI)	*P*
**FLI**				
T1	Reference	–	Reference	–
T2	1.43 (1.05–1.94)	0.022	1.05 (0.57–1.92)	0.885
T3	2.44 (1.84–3.24)	< 0.001	1.95 (1.13–3.36)	0.016
Age (per 1 year)	1.01 (1.00–1.02)	0.099	1.01 (0.99–1.03)	0.531
FPG (per 1 mg/dL)	1.13 (1.12–1.14)	< 0.001	1.12 (1.10–1.14)	< 0.001
Smoking habit	1.68 (1.41–2.01)	< 0.001	1.42 (0.92–2.20)	0.115
Alcohol drinking habit	0.60 (0.50–0.71)	< 0.001	0.82 (0.55–1.24)	0.356
Family history of DM	0.90 (0.65–1.24)	0.510	0.77 (0.39–1.53)	0.451
Hypertension	1.11 (0.91–1.36)	0.293	1.08 (0.68–1.71)	0.758
Dyslipidemia	1.52 (1.13–2.04)	0.006	2.31 (1.20–4.46)	0.013
	AIC = 7616		AIC = 1732	

When further divided by the absence and presence of impaired fasting glucose (IFG), HRs after adjustment of covariates gradually increased from T1 to T3 and from the absence to presence of IFG in both male and female subjects (Table 5). Even in the absence of IFG, HRs in the T3 group were significantly higher than those in the T1 group in both male and female subjects.

**Table 5:** Multivariable Cox proportional hazard regression analyses for new onset of DM in tertiles of FLI in the absence and presence of IFG.

	n	HR (95% CI)	*P*
**Male subjects**	7925		
IFG (−)			
T1	2390	Reference	-
T2	2203	1.69 (1.06–2.68)	0.026
T3	1952	3.99 (2.63–6.04)	< 0.001
IFG (+)			
T1	248	12.8 (7.74–21.2)	< 0.001
T2	442	16.6 (10.8–25.6)	< 0.001
T3	690	30.0 (20.2–44.6)	< 0.001
**Female subjects**	4365		
IFG (−)			
T1	1430	Reference	–
T2	1391	1.19 (0.60–2.39)	0.618
T3	1270	2.19 (1.16–4.14)	0.016
IFG (+)			
T1	28	9.51 (2.72–33.2)	< 0.001
T2	64	12.9 (5.61–29.4)	< 0.001
T3	182	25.7 (13.7–48.1)	< 0.001

*CI* confidence interval, *DM* diabetes mellitus, *FLI* fatty liver index, *HR* hazard ratio, *IFG* impaired fasting glucose.

IFG was defined as fasting plasma glucose ≥ 100 mg/dL.

The model was adjusted for age, smoking habit, alcohol drinking habit, family history of DM, hypertension and dyslipidemia.

## Discussion

The present study showed that a high level of FLI was significantly associated with the risk of DM development during a 10-year period in both male and female subjects. It has been reported that FLI, originally developed for diagnosis of NAFLD, is associated with several metabolic diseases^26^. Furthermore, FLI has been proposed to be a marker for incidence of type 2 DM (Supplementary Table S1)^18–25^. Earlier studies showed an association between a high FLI level (≥ 60–70) as diagnosis of NAFLD and incidence of DM in logistic regression analyses, but the cumulative incidence of DM was not analyzed^18–20^. Several studies also showed a high FLI level (FLI ≥ 60) was associated with the cumulative incidence of DM in Cox proportional regression analyses using a relatively small number of subjects (n = 1142–1922)^21,22,24^. Furthermore, in only one study using 389 subjects with prediabetes defined as FPG level of 110–125 mg/dL, the association of DM development with level of FLI as a continuous variable was examined by Cox proportional regression analysis^23^. The present study showed that the risk of DM development continuously increased with a higher FLI at baseline in both male and female subjects in multivariable Cox proportional hazard models with a restricted cubic spline using a large number of subjects (n = 12,290, male/female: 7935/4365).

Since there is a sex difference in components of FLI calculation, including BMI, WC, triglycerides and γ-GTP, being higher in male subjects than in female subjects^27–30^, it is necessary to analyze the FLI value divided by sex. In fact, there was a significant sex difference in FLI level in the present study as well as in a previous study^16^. Definitions of FLI ≥ 60 as NAFLD and FLI < 30 as non-NAFLD have been used in several studies^20–24^. However, it has been reported that cutoff levels of FLI for diagnosis of NAFLD seem to be lower in Asians than in Europeans: FLI ≥ 30 in China^17^ and FLI ≥ 60 in Italy^14^, in which subjects were not divided by sex in the both studies^14,17^. Although there have been no studies about the validation of FLI in other races including African Americans and Hispanics, it has been reported that the prevalence of NAFLD is higher in Hispanics than in non-Hispanic whites and African Americans^31^. These findings suggest that there might be racial and sex differences in cutoff levels of FLI for diagnosis of NAFLD. In the present study, the optimal cutoff value of FLI to predict incidence of DM was higher in male subjects than in female subjects (FLI: 56.6 vs. 23.7) (Fig. 2A,B). Therefore, instead of the conventional division of FLI (FLI ≥ 60 and < 30), we analyzed HRs in subgroups according to tertiles of FLI level at baseline in both sexes in the present study (Table 4).

The level of FPG at baseline was found to be a strong predictor of new onset of DM in the present study. The ability of FLI at baseline to predict new onset of DM was comparable to that of FPG in both male and female subjects. When further divided by the absence and presence of IFG in tertiles of FLI level, HRs after adjustment of confounders gradually increased from T1 to T3 and from the absence to presence of IFG in both male and female subjects (Table 5). Similar results were obtained in a previous study by Hirata et al., though the number of subjects was relatively small (n = 4439, male/female: 1498/2,941)^25^. HRs of DM development in the T2 and T3 groups in the presence of IFG in the present study (male/female: 16.6–30.0/12.9–25.7) were higher than those in the previous study (male/female: 3.4–5.2/5.9–6.2)^25^. Furthermore, HRs of DM development in the T3 group in the absence of IFG were significantly higher than those in the T1 group of both sexes in the present study, but there was no significant difference in HRs in the non-IFG group of both sexes in the previous study^25^. Possible reasons for the difference were the number of study subjects (12,290 vs. 4439) and length of the follow-up period (mean: 9.5 vs. 3.0 years). Therefore, the results of the present study may accurately reflect the risk of DM development.

It has been reported that insulin resistance with visceral obesity causes compensatory hyperinsulinemia, leading to the development of NAFLD^32^. Furthermore, fat accumulation in the liver contributes to an increase of glucose production and high serum free fatty acid flux, which is caused by impaired insulin action^33^. Conversely, insulin-resistant fatty liver overproduces glucose and very low-density lipoprotein, leading to exhaustion of the pancreatic β cell reserve and subsequent development of DM^32^. Since FLI has been reported to be strongly associated with reduced insulin sensitivity assessed by the euglycemic hyperinsulinemic clamp method^34^, a possible mechanism for the association between FLI and DM development is insulin resistance. As another possibility of the mechanism, several hepatokines, secretory molecules from the liver, mediate the relationship between NAFLD and the development of DM. A steatotic and inflamed liver has been reported to secrete several hepatokines, including fetuin-A^35^, fibroblast growth factor 21^36,37^, selenoprotein P^38^ and xanthine oxidase^39–41^, which are known to be have endocrine functions at extrahepatic sites to cause insulin resistance and other adverse effects on glucose homeostasis.

The present study has some limitations. First, FLI was used as a surrogate marker for NAFLD because of the absence of liver biopsy as a gold standard for diagnosis of NAFLD and imaging examinations including ultrasonography, computed tomography and magnetic resonance spectroscopy^12^. However, it has recently been reported that FLI can predict NAFLD and overcome the limitation of diagnosis based on abdominal ultrasonography^42^. Second, the presence of hepatitis B and hepatitis C was unknown at baseline, though the prevalence of hepatitis B (0.63%) and hepatitis C (0.49%) was reported to be relatively low in the Japanese population^43,44^. Third, accurate information on alcohol consumption was not obtained in the present study. However, when subjects who had an alcohol drinking habit were excluded from analyses, most of the results were similar (Supplementary Tables S3, S4). Fourth, the possibility of selection bias in the samples cannot be excluded since the study subjects were urban residents who received annual health checkups in a single clinic. Finally, the relationship between change in FLI level and new onset of DM was not investigated in the present study, and this needs to be examined in the future.

In conclusion, an elevated FLI level can predict the development of DM during a 10-year period in a general population of both sexes. The pathophysiology underlying the association between factors that influence FLI level and DM development needs to be addressed in future basic and clinical studies.

## Methods

### Study subjects

A total of 28,990 subjects who received annual medical checkups at Keijinkai Maruyama Clinic, Sapporo, Japan in 2006 were enrolled in this registry^45,46^. A flow chart of the study subjects is shown in Fig. 1. Exclusion criteria were the diagnosis of DM at baseline and absence of data for WC, BMI and laboratory data including FPG, HbA1c, triglycerides and γGTP at baseline. After prespecified exclusion, a total of 12,290 subjects (male/female: 7925/4365) who received annual health examinations at least once from 2007 to 2015 were finally recruited in the present study. The study was performed with the approval of the institutional ethical committee of Sapporo Medical University (Numbers: 29-2-64, 30-2-32) and conformed to the principles of the Declaration of Helsinki. Written informed consent was obtained from the recruited subjects.

### Measurements

Blood pressure measurements, medical examinations and samplings of blood were performed after an overnight fast. Body height and weight were measured, and BMI was calculated as body weight in kilograms divided by height in meters squared. HbA1c level was presented as the National Glycohemoglobin Standardization Program (NGSP) equivalent value. eGFR was calculated by an equation for Japanese: eGFR (mL/min/1.73 m^2^) = 194 × serum creatinine^(−1.094)^ × age^(−0.287)^ × 0.739 (if female)^47^. A self-administered questionnaire survey was performed to obtain information on current smoking habit, alcohol drinking habit (≥ 3 times/week), family history of DM, and use of drugs for hypertension, dyslipidemia and DM.

DM was diagnosed in accordance with the guidelines of the American Diabetes Association^48^: self-reported use of anti-diabetic drugs, FPG ≥ 126 mg/dL or HbA1c ≥ 6.5%. IFG was defined as FPG ≥ 100 mg/dL^48^. Hypertension was diagnosed as self-reported use of anti-hypertensive drugs, systolic blood pressure ≥ 140 mmHg or diastolic blood pressure ≥ 90 mmHg. Dyslipidemia was diagnosed as self-reported use of anti-dyslipidemic drugs, low-density lipoprotein cholesterol ≥ 140 mg/dL, triglycerides ≥ 150 mg/dL or high-density lipoprotein cholesterol < 40 mg/dL.

### Fatty liver index

FLI was calculated using the following formula^14^: FLI = [e^(0.953 × ln(triglycerides) + 0.139 × BMI + 0.718 × ln(γGTP) + 0.053 × WC − 15.745)^]/[1 + e^(0.953 ×ln(triglycerides) +0.139 × BMI + 0.718 × ln(γGTP) + 0.053 × WC − 15.745)^] × 100.

### Statistical analysis

Numeric variables are expressed as means ± SD for normally distributed parameters or medians (interquartile ranges) for skewedly distributed parameters. The distribution of each parameter was tested for its normality using the Shapiro–Wilk W test. Comparison between two groups was done with Mann–Whitney's U test. Intergroup differences in demographic parameters were examined by the chi-square test. For detecting significant differences between data in multiple groups, one-way analysis of variance was used. The ability of FLI or FPG at baseline to predict new onset of DM was investigated using receiver operating characteristic (ROC) curves. The area under curve (AUC) was calculated, and cut-off values of FLI and FPG were obtained by the Youden index^49^. The relationship between FLI and hazard ratio (HR) for the development of DM after adjustment of confounders including age, FPG, habits of alcohol drinking and current smoking, family history of DM and diagnosis of hypertension and dyslipidemia at baseline was analyzed by a multivariable Cox proportional hazard model with a restricted cubic spline. HRs and 95% confidence intervals in three subgroups according to tertiles of FLI level at baseline (T1–T3) in both males and females were calculated by adjustment of the covariates. HRs for new onset of DM among the T1–T3 groups in the absence and presence of IFG were also analyzed. A *P* value of < 0.05 was considered statistically significant. All data were analyzed by using EZR^50^ and R: A Language and Environment for Statistical Computing version 3.6.1 (R Core Team, R Foundation for Statistical Computing, Vienna, Austria, 2019, https://www.R-project.org).

## Supplementary information

Supplementary Tables.

## Data Availability

The datasets analyzed during the current study are available from the corresponding author on reasonable request.
