# Supplementary material for: High level of fatty liver index predicts new onset of diabetes mellitus during a 10-year period in healthy subjects
Source: Sci Rep. 2021 Jun 18;11:12830. doi: 10.1038/s41598-021-92292-y (PMC8213804; doi:10.1038/s41598-021-92292-y)
Supplement: Supplementary file 1 — Supplementary Tables. [file 41598_2021_92292_MOESM1_ESM.pdf]

Supplementary materials

High level of fatty liver index predicts new onset of diabetes mellitus during a 10-year period in healthy subjects

Yukimura Higashiura[1*], Masato Furuhashi[1*]†, Marenao Tanaka[1*], Satoko Takahashi[1], Masayuki Koyama[1,2], Hirofumi Ohnishi[1,2], Keita Numata[3], Takashi Hisasue[3], Nagisa Hanawa[3], Norihito Moniwa[1], Kazufumi Tsuchihashi[1], Tetsuji Miura[1]
**The first three authors equally contributed to this work.*

[1]Department of Cardiovascular, Renal and Metabolic Medicine, Sapporo Medical University School of Medicine; [2]Department of Public Health, Sapporo Medical University School of Medicine, Sapporo, Japan; [3]Department of Health Checkup and Promotion, Keijinkai Maruyama Clinic, Sapporo, Japan

Supplementary Table S1

Supplementary Table S1. Summary of studies regarding the association between the development of diabetes mellitus and FLI level.

				Number	Age (years)	Duration		Regression analysis			Divided	Refs
Authors	Year	Country	Subjects	All (M/F)	(range/mean)	(years)	Subgroups of FLI		by A*	by B*	by sex	
Balkau B, et al.	2010	France	GP	3,811 (1,861/1,950)	30-65 / 47	9	< 20, 20-69, ≥ 70	Logistic	(+)	(-)	(+)	18
Jung CH, et al.	2013	Korea	GP	7,860 (5,154/2,706)	20-83 / 49	4	< 20, 20-59, ≥ 60	Logistic	(+)	(-)	(-), Adjusted	19
Yadav D et al.	2016	Korea	GP	2,784 (944/1,840)	40-70 / 55	2.6	< 30, 30-59, ≥ 60	Logistic	(+)	(-)	(-), Adjusted	20
Jager S, et al.	2015	Germany	GP	1,922 (707/1,215)	? / 49	8	< 30, 30-59, ≥ 60	Cox proportional	(+)	(-)	(+)	21
Franch-Nadal J, et al.	2018	Spain	Prediabetes	1,142 (574/568)	30-74 / ?	2.8	< 30, 30-59, ≥ 60	Cox proportional	(+)	(-)	(-), Adjusted	22
Wargny M, et al.	2019	France	Prediabetes	389 (252/137)	? / 58	3.9	< 30, 30-59, ≥ 60	Cox proportional	(-)	(+)	(-), Adjusted	23
Olubamwo OO, et al.	2019	Finland	Male only	1,792 (1792/ -)	42-60 / 53	18.8	< 30, 30-59, ≥ 60	Cox proportional	(+)	(-)	NA	24
Hirata A, et al.	2018	Japan	GP	4,439 (1,498/2,941)	40-74 / 65	3	Tertile (+/- IFG)	Cox proportional	(+)	(-)	(+)	25
Higashiura Y, et al	2021	Japan	GP	12,290 (7,925/4,365)	19-96 / 48	9.5	Tertile (+/- IFG)	Cox proportional	(+)	(+), Cubic spline	(+)	This study

FLI, fatty liver index; GP, general population; IFG, impaired fasting glucose; NA, not applicable.
A*: analysis using categorized subgroups of FLI. B*: analysis using FLI level as a continuous variable.

Supplementary Table S2

Supplementary Table S2. Characteristics of the enrolled and excluded subjects.

	Total<br>n = 28,890	Enrolled<br>n = 12,290	Excluded<br>n = 16,600	P
Age (years)	45 ± 13	48 ± 10	42 ± 14	<0.001
Sex (male/female)	17888 (61.9)	7,925 (64.5)	9,963 (60.0)	<0.001
Body mass index	22.9 ± 3.5	23.2 ± 3.4	22.7 ± 3.7	<0.001
Waist circumference (cm)	82.4 ± 9.8	83.4 ± 9.2	81.3 ± 10.3	<0.001
Systolic blood pressure (mmHg)	116 ± 17	117 ± 16	115 ± 17	<0.001
Diastolic blood pressure (mmHg)	73 ± 11	74 ± 11	73 ± 11	<0.001
Smoking habit	10,235 (35.4)	4,231 (34.4)	6,004 (36.2)	<0.001
Alcohol drinking habit	11,817 (40.9)	5,634 (45.8)	6,183 (37.2)	<0.001
Family history				
Diabetes mellitus	5,041 (17.4)	2,115 (17.2)	2,926 (17.6)	0.49
Comorbidity				
Hypertension	4,351 (15.1)	2,068 (16.8)	2,283 (13.8)	<0.001
Dyslipidemia	6,194 (21.4)	2,720 (22.1)	3,474 (20.9)	0.006
Biochemical data				
Albumin (g/dL)	4.4 ± 0.2	4.4 ± 0.2	4.4 ± 0.2	0.307
eGFR (mL/min/1.73m^2)	85.2 ± 15.3	84.5 ± 14.6	86.0 ± 16.0	<0.001
Uric acid (mg/dL)	5.4 ± 1.4	5.5 ± 1.4	5.4 ± 1.4	<0.001
FPG (mg/dL)	93 ± 20	90 ± 9	95 ± 25	<0.001
Hemoglobin A1c (%)	5.4 ± 0.7	5.2 ± 0.4	5.7 ± 1.1	<0.001
AST (U/L)	23 (18-25)	23 (18-26)	23 (17-25)	<0.001
ALT (U/L)	26 (14-30)	26 (15-31)	25 (14-29)	<0.001
γGTP (U/L)	47 (18-52)	50 (19-56)	44 (17-49)	<0.001
Total cholesterol (mg/dL)	202 ± 35	204 ± 34	200 ± 35	<0.001
LDL cholesterol (mg/dL)	119 ± 32	122 ± 31	117 ± 32	<0.001
HDL cholesterol (mg/dL)	62 ± 16	61 ± 16	62 ± 16	<0.001
non-HDL cholesterol (mg/gL)	141 ± 37	144 ± 35	138 ± 37	<0.001
Triglycerides (mg/dL)	111 (60-133)	113 (63-136)	109 (57-131)	<0.001
FLI	28.4 (6.2-45.7)	30.1 (7.9-48.2)	26.4 (4.9-42.2)	<0.001

Variables are expressed as number (%), means ± SD or medians (interquartile ranges).
AST, aspartate aminotransferase; ALT, alanine aminotransferase; eGFR, estimated glomerular filtration rate; FLI, fatty liver index; FPG, fasting plasma glucose; γGTP; γ-glutamyl transferase; HDL, high-density lipoprotein; LDL, low-density lipoprotein.

Supplementary Table S3

Supplementary Table S3. Multivariable Cox proportional hazard analyses for new onset of DM in tertiles of FLI in subjects without alcohol drinking habit.

	Male (n = 3,433)		Female (n = 3,223)	
	HR (95% CI)	P	HR (95% CI)	P
FLI				
T1	Reference	-	Reference	-
T2	2.00 (1.20-3.35)	0.008	0.98 (0.49-1.98)	0.956
T3	3.57 (2.21-5.78)	< 0.001	1.87 (1.00-3.49)	0.049
Age (per 1 year)	1.03 (1.01-1.04)	0.003	1.01 (0.99-1.03)	0.442
FPG (per 1 mg/dL)	1.12 (1.10-1.13)	< 0.001	1.11 (1.09-1.13)	< 0.001
Smoking habit	1.58 (1.19-2.09)	0.002	1.31 (0.75-2.31)	0.347
Family history of DM	0.73 (0.44-1.18)	0.194	0.79 (0.36-1.71)	0.548
Hypertension	0.96 (0.69-1.33)	0.796	1.13 (0.66-1.93)	0.678
Dyslipidemia	2.12 (1.36-3.30)	< 0.001	2.14 (1.04-4.43)	0.039
	AIC = 3,064		AIC = 1,345	

AIC, Akaike's information criterion; CI, confidence interval; DM, diabetes mellitus; FLI, fatty liver index; FPG, fasting plasma glucose; HR, hazard ratio.

Supplementary Table S4

Supplementary Table S4. Multivariable Cox proportional hazard regression analyses for new onset of DM in tertiles of FLI in the absence and presence of IFG in subjects without alcohol drinking habit..

	n	HR (95% CI)	P
Male subjects	3,433		
IFG (-)			
T1	1,212	Reference	-
T2	961	1.79 (0.96-3.34)	0.068
T3	792	4.86 (2.78-8.50)	< 0.001
IFG (+)			
T1	109	8.99 (4.21-19.2)	< 0.001
T2	148	16.6 (8.96-30.6)	< 0.001
T3	211	28.8 (16.6-49.9)	< 0.001
Female subjects	3,223		
IFG (-)			
T1	1,106	Reference	-
T2	1,008	1.37 (0.61-3.04)	0.444
T3	918	2.60 (1.24-5.42)	0.011
IFG (+)			
T1	20	15.3 (4.19-55.7)	< 0.001
T2	46	13.8 (5.17-36.7)	< 0.001
T3	125	24.5 (11.6-51.7)	< 0.001

CI, confidence interval; DM, diabetes mellitus; FLI, fatty liver index; HR, hazard ratio; IFG, impaired fasting glucose.
IFG was defined as fasting plasma glucose ≥ 100 mg/dL.
The model was adjusted for age, smoking habit, alcohol drinking habit, family history of DM, hypertension and dyslipidemia.
